# Multiparameter resonant imaging for studying cell interactions

**DOI:** 10.1038/s41377-018-0032-y

**Published:** 2018-07-25

**Authors:** José Juan-Colás, Thomas F Krauss

**Affiliations:** 1 Department of Physics, The University of York, Heslignton, YO10 5DD UK; 2 Department of Electronic Engineering, The University of York, Heslignton, YO10 5DD UK

The development of optical microscopy techniques has provided major insights into the machinery of life, from molecules to cells to tissues and organs. Fluorescent techniques have been particularly successful in this respect by elucidating biological pathways and helping us understand the complex relationships that underpin all living organisms. For example, the first use of fluorescence as an analytical tool in 1864^1^ paved the way for the study of biological entities and interactions, but it was not until nearly 60 years later, in the 1930s, that fluorophores were first employed to perform biological investigations into specific tissue components and bacteria that do not autofluoresce^2^. These advances led to the first labeling of an antibody with a visible label in 1941^3^, which gave birth to the field of immunofluorescence and allowed us obtain unprecedented insight into antibody structure.

Nevertheless, fluorescence techniques require the addition of labels, which complicate the procedure and may interfere with the very pathways that we are trying to understand^4,5^. As a result, new methods have been developed that do not require the addition of fluorescent labels but use photonic resonances to enhance the weak interaction between electromagnetic fields and the biological objects of interest. Moreover, it is important to detect multiple parameters in parallel since information about the density, adhesion, or motility of a living body and properties such as the stiffness and impedance of biological objects all provide insights into our understanding of biological pathways. An important step toward this goal of multiparameter characterization has now been taken with the introduction of a resonant label-free microscopy technique that offers insights into both the density of cells and their adhesion to a substrate. The technique exploits guided mode resonances in photonic crystals and is termed photonic resonator outcoupler microscopy (PROM)^6^. PROM can detect changes in both the phase and amplitude of the photonic resonance that is caused by the presence of a cell. The change in phase is related to the optical density, i.e., it indicates the presence of cellular matter, while the change in amplitude is related to the shape of the cell, which indicates adhesion to the surface.

Related techniques include surface plasmon resonance microscopy (SPRM)^7^, photonic crystal enhanced microscopy (PCEM)^8^ and elastic resonator interference stress microscopy (ERSIM)^9^. SPRM and PCEM sense changes in the refractive index (**n**) within the first ~200 nm from the substrate and have demonstrated the capability of monitoring cell attachment^10^ and protein diffusion at the cell membrane level^11^, while ERSIM detects adhesion and, more generally, forces that are exerted by the cell on the surface^8^. Deeper, complementary biological information can be retrieved with PROM by examining the refractive index variations more carefully; PROM is able to disentangle changes in the refractive index from changes in scattering, which predominantly occur at the edges of an object^6^. The authors explore this effect in the context of stem cell imaging to identify whether these changes are associated with standard forces from cell-matrix adhesions or with specific aggregations of proteins at the cell membrane level. Such aggregations are known as focal adhesions (FAs), and they regulate the engagement of the cells with their environment as well as their motility (Fig. 1).

**Fig. 1:**
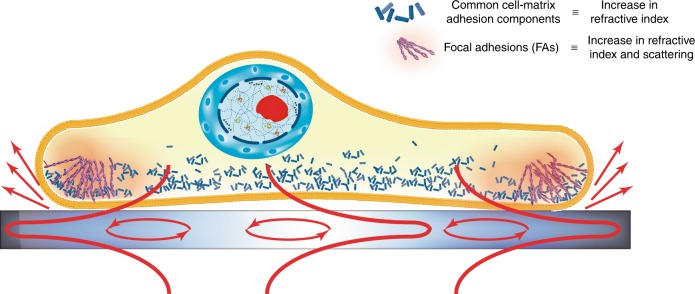
Illustration of the cross-section of a cell attached to a surface. At the cell-surface interface, particular cell membrane components are located at the outer regions of the cell membrane to regulate engagement and motility. These components agregate at the edges of a cell and increase light scatterring, thereby reducing the amplitude of the photonic resonance, compared to the common cell-matrix adhesions, which impact on its phase

Standard cell-matrix adhesion shows a significant variation in the refractive index but has a low impact on scattering. In contrast, FAs exhibit notable variations in the refractive index and a strong increase in scattering. Therefore, the PROM technique is able to identify the locations of these FAs within the cell membrane and yields results that are closely correlated with fluorescence-based measurements without the need for fluorescent dyes and the inconvenience they entail. Therefore, the PROM technique is a valuable label-free tool for performing dynamic, long-term and quantitative imaging of cell-surface interactions on a micrometer scale^12^.

This approach for disentangling changes in refractive index from changes in scattering, which is exemplified here in the context of stem cell imaging, can open new avenues for interpreting the information provided by label-free imaging and offers novel insights into biological processes. If scattering can be related to molecular density^6^, it can also be employed to estimate molecular conformation, as they are highly linked in some biological processes^13^. Therefore, by interpreting the effect of scattering on some biological systems, it is possible to further characterize the ongoing biological processes, as biological conformation determines biological function.

## References

[1] Stokes GG (1864). XXXIV.—On the application of the optical properties of bodies to the detection and discrimination of organic substances. J. Chem. Soc..

[2] Haitinger M, Hamperl H (1933). Die anwendung des fluoreszenzmikroskops zur untersuchung tierischer gewebe. Ztschr. mikro.-anat. Forsch..

[3] Coons AH, Creech HJ, Jones RN (1941). Immunological properties of an antibody containing a fluorescent group. Exp. Biol. Med..

[4] Genty B, Briantais JM, Baker NR (1989). The relationship between the quantum yield of photosynthetic electron transport and quenching of chlorophyll fluorescence. Biochim. Biophys. Acta.

[5] Terai T, Nagano T (2013). Small-molecule fluorophores and fluorescent probes for bioimaging. Pflug. Arch..

[6] Zhuo, Y. et al. Quantitative analysis of focal adhesion dynamics using photonic resonator outcoupler microscopy (PROM). *Light Sci. Appl*. 10.1038/s41377-018-0027-8 (2018). 10.1038/s41377-018-0001-5 PMC6020849 29963322

[7] Campbell CT, Kim G (2007). SPR microscopy and its applications to high-throughput analyses of biomolecular binding events and their kinetics. Biomaterials.

[8] Zhuo Y, Cunningham BT (2015). Label-free biosensor imaging on photonic crystal surfaces. Sensors.

[9] Kronenberg NM (2017). Long-term imaging of cellular forces with high precision by elastic resonator interference stress microscopy. Nat. Cell Biol..

[10] Lidstone EA (2011). Label-free imaging of cell attachment with photonic crystal enhanced microscopy. Analyst.

[11] Wang W (2012). Label-free measuring and mapping of binding kinetics of membrane proteins in single living cells. Nat. Chem..

[12] Triggs GJ (2015). Spatial resolution and refractive index contrast of resonant photonic crystal surfaces for biosensing. IEEE Photonics J..

[13] Juan-Colás J, Krauss TF, Johnson SD (2017). Real-time analysis of molecular conformation using silicon electrophotonic biosensors. ACS Photonics.

